# Surgical treatment of subchondral osteonecrosis of the humeral head: A case report and literature review

**DOI:** 10.1097/MD.0000000000034389

**Published:** 2023-08-04

**Authors:** Yongsheng Liu, Jia Zhong, Zhaowei Jiang, Duo Shen, Daohong Zhao

**Affiliations:** a Department of Orthopaedics, The Second Affiliated Hospital of Kunming Medical University, China; b Department of Orthopaedics, The People’s Hospital of XiShuangBanBa State, China; c Department of Orthopaedics, The People’s Hospital of Dehong State, China; d Department of Orthopaedics, The People’s Hospital of Longchuan County, China.

**Keywords:** cartilage transplantation, case report, etiology, necrosis of the humeral head, surgical treatment

## Abstract

Osteonecrosis of the humeral head is an uncommon subchondral bone disease with many etiologies, and there is currently no definite evidence to support an optimal surgical treatment plan. We report a case of surgical treatment of left humeral head necrosis. To the best of our knowledge, this is the youngest patient with non-drug-induced humeral head necrosis and the largest collapsed area.

**Case presentation:**

The case involved a 16-year-old male who injured his left shoulder 1 year ago. The patient was admitted to the hospital because of shoulder pain after activity in the year following the injury. During the physical examination, the left glenohumeral joint space was tender, the pain was obvious when the shoulder joint was rotated and squeezed, and the active and passive range of motion was normal. X-ray, magnetic resonance imaging, and computed tomography + 3D computed tomography scans all showed subchondral osteonecrosis of the left humeral head. Left humeral head lesion removal and autologous osteochondral transplantation were performed, and the patient was followed up.

**Conclusion:**

Non-drug-induced humeral head necrosis is rare. Autologous osteochondral transplantation is currently one of the most mature and effective treatment methods. The short-term curative effect in this patient is satisfactory, but the patient is young and has a large collapsed area, so long-term follow-up is worthwhile.

## 1. Introduction

Osteonecrosis of the humeral head is an uncommon subchondral bone disease. Although it has been reported in the literature that its incidence ranks second among nontraumatic osteonecroses after femoral head necrosis,^[1]^ only a few studies have been published. The etiology is classified into 3 categories: traumatic, nontraumatic, and idiopathic. Among nontraumatic factors, corticosteroids are the most common cause of osteonecrosis.^[2]^ Necrosis can manifest as osteochondritis dissecans, usually in the knee, ankle, and elbow and rarely in the shoulder (involving the humeral head or glenoid). At present, according to the Cruess grading system,^[3]^ surgical treatment is recommended for Stages III to V necrosis, but surgery is controversial because definite evidence for an optimal surgical treatment plan is lacking. We admitted a young patient with necrosis of the left humeral head for surgery. To the best of our knowledge, this is the youngest patient with non-drug-induced humeral head necrosis and the largest collapsed area.

## 2. Case presentation

**Figure 1.**
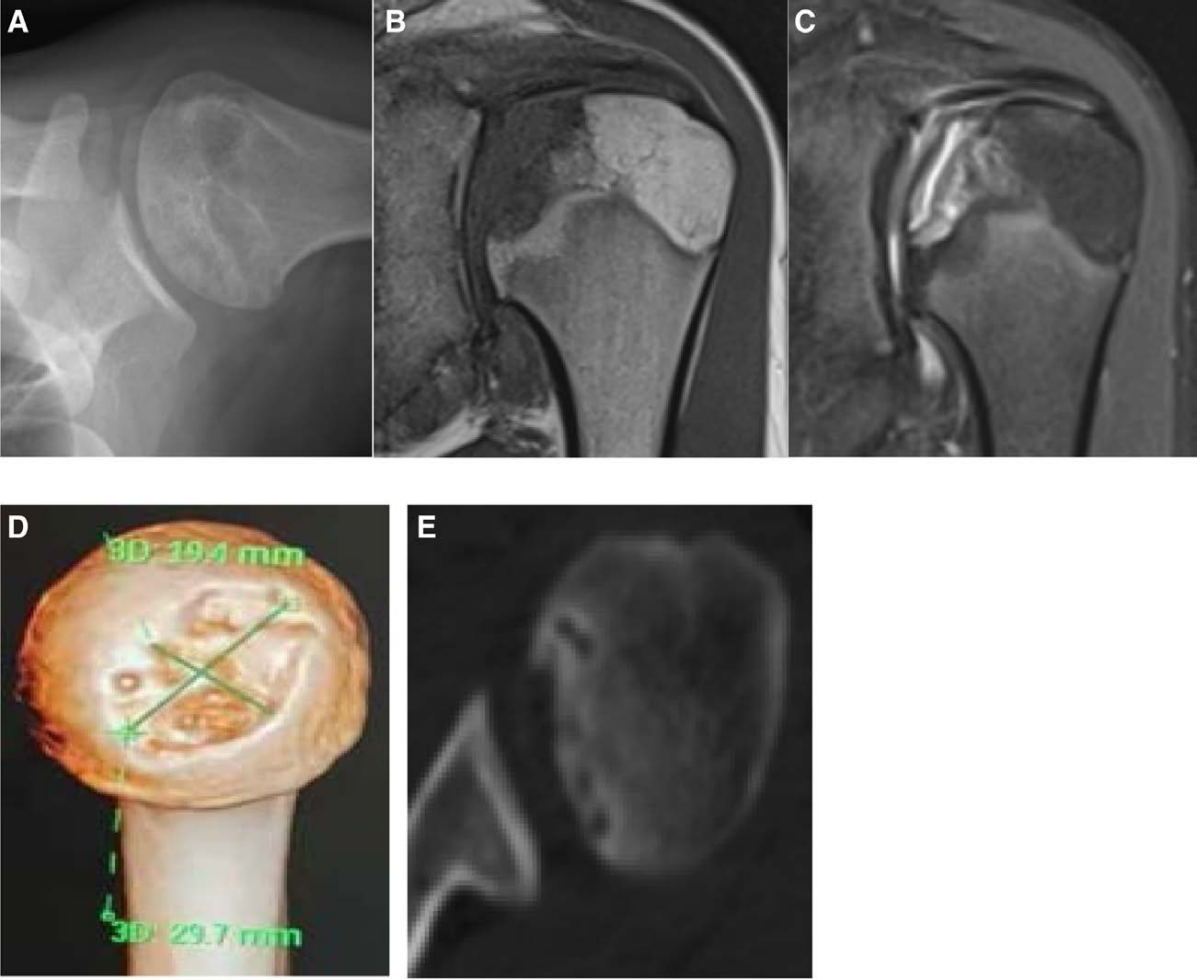
(A) X-ray AP of pre-operation. (B) MRI T1-weighted image. (C) MRI T2-weighted image. (D) 3-dCT scan. (E) CT scan cross section. CT = computed tomography, MRI = Magnetic resonance image.

This case involved a 16-year-old male patient. His left shoulder was injured while playing basketball 1 year ago. After the injury, he suffered swelling and pain, with limited movement and no shoulder deformity. However, the patient did not go to the hospital for diagnosis and treatment. After approximately 1 month, the pain had disappeared when the patient was at rest but reappeared after activity. The pain was positively correlated with the activity volume, degree and time, but the patient still insisted on playing basketball in the year after the injury. The patient was referred to our hospital for diagnosis and treatment due to recurrent pain symptoms. The patient had no history of long-term steroid use, alcohol abuse, systemic lupus erythematosus or rheumatoid disease. During the physical examination, the patient had no obvious deformity of the left shoulder, the pain was obvious when the shoulder joint was rotated and squeezed, and the active and passive range of motion was normal. There was no rotator cuff injury or shoulder instability. X-ray showed a slightly flattened left humeral head, a mixed shadow of high and low density, and a crescent sign (Fig. 1A). Magnetic resonance T1-weighted imaging showed depression of the left humeral head, decreased local bone signal intensity, and articular cartilage detachment. T2-weighted imaging showed a bone depression under the articular surface, mixed signal shadows, articular cartilage detachment, and articular cartilage discontinuity (Fig. 1B and C). Computed tomography showed an irregular shape of the left humeral head, a bony depression on the articular surface, and multiple cystic bone resorption under the articular surface. A 3D computed tomography (CT) scan showed oblong necrosis of bone on the articular surface of the left humeral head, irregular depression, and a collapsed area of approximately 576 mm^2^ (29.7 mm × 19.4 mm) (Fig. 1D and E). All results suggested subchondral osteonecrosis of the left humeral head.

**Figure 2.**
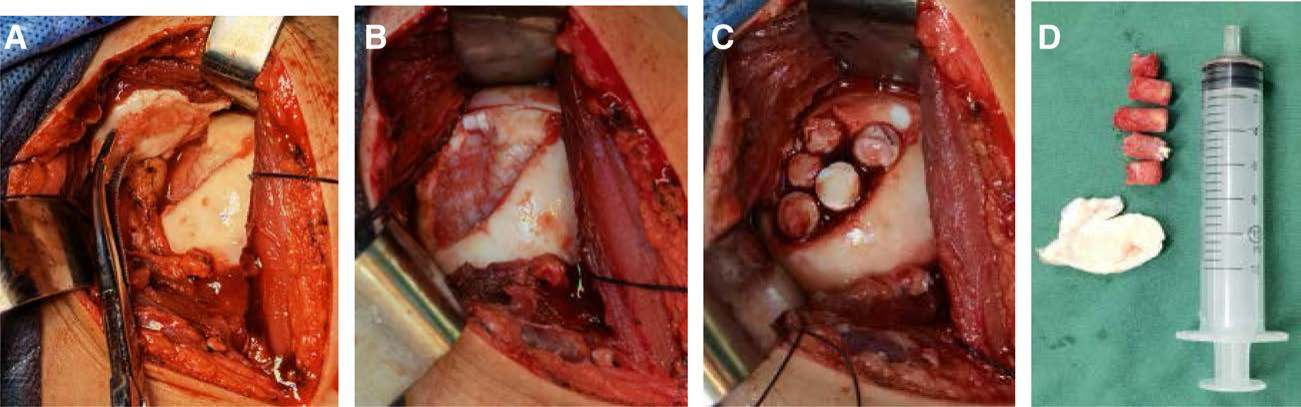
(A) On the medial articular surface of the left humeral head, the cartilage surface is discontinuous, large pieces of cartilage are peeled off from the subchondral bone, and cartilage degeneration. (B) Subchondral bone sclerosis, cystic degeneration, and concave bone surface. (C) Autologous cartilage column was implantated disease injury-area, repaired cartilage articular surface. (D) Necrotic stripped cartilage and autologous cartilage column.

The patient was initially diagnosed with subchondral osteonecrosis of the left humeral head and underwent surgical treatment with general anesthesia in the beach chair position. The left shoulder surgical area and the left knee joint were cleaned with disinfection towels. The left deltoid muscle pectoralis major space approach was taken; the incision was approximately 8 cm, and the cephalic vein was protected at each layer. The left shoulder joint cavity was exposed; the joint fluid was clear, the humeral head cartilage was partially free, and the size was 10 mm × 30 mm. Next, the subchondral bone was exposed, the free cartilage was removed, and the surrounding cartilage was freshened. Cartilage transplantation tools (Arthrex) were used. The necrotic bone was removed, and a small incision was made in the left knee joint. Three 8 mm osteochondral columns were taken from the upper part of the femoral trochlea to be transplanted in the defect of the humeral head, and two 8 mm periosteal bone columns were taken from the humeral head. At the defect, the surface was flush with the surrounding cartilage, and the stability of the grafted osteochondral column was explored. The incision was closed layer by layer after the subscapularis tendon was sutured and the drainage tube was indwelled (Fig. 2).

**Figure 3.**
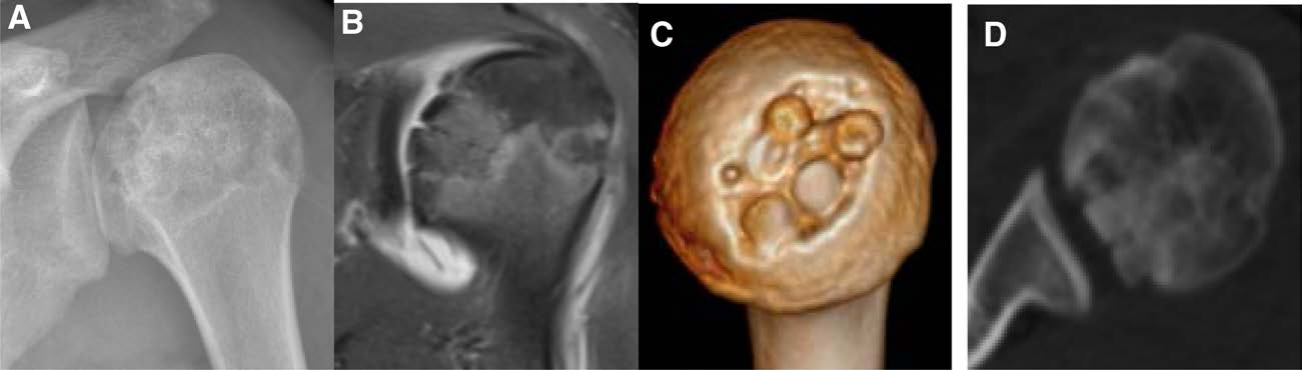
The postoperative X-ray, MRI T2-weighted image, and CT + 3D CT scan showed that the shape of the left humeral head was significantly improved compared with preoperative, the necrosis was removed, autologous cartilage was implanted, and the articular surface was repaired. CT = computed tomography, MRI = Magnetic resonance image.

**Figure 4.**
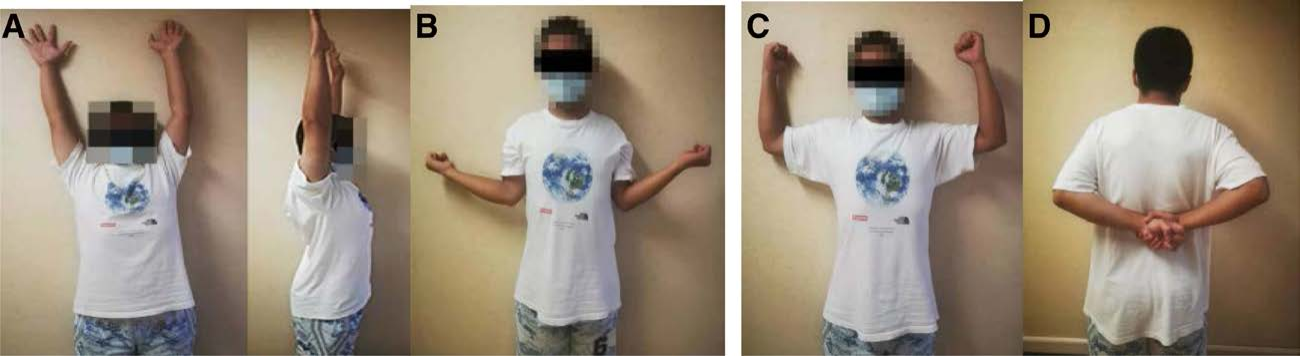
The patient’s shoulder joint function: (A) forward flexion, (B) external rotation, (C) abduction 90° external rotation, and (D) internal rotation.

After surgery, the patient was given a triangular scarf for protection. On the second day, the patient underwent X-ray, magnetic resonance imaging and CT + 3D CT scans for imaging evaluation and follow-up (Fig. 3). One week after the operation, the patient was instructed to perform shoulder rehabilitation exercises. One month after the surgery, the patient was satisfied with shoulder joint function (Fig. 4) and returned to school. Written informed consent was obtained from the patients mothor for publication of this case report details.

## 3. Discussion

In this manuscript, we describe a rare clinical case of surgical treatment of subchondral osteonecrosis of the left humeral head. The patient in this case is the youngest patient to date with non-drug-induced humeral head necrosis, and the size of the collapsed area is the largest.

The humeral head is supplied by abundant anastomotic arteries, among which the arcuate artery, which is the ascending anterolateral branch of the anterior circumflex artery, is considered to be the most important supplying vessel.^[4]^ When traumatic factors (fractures) or nontraumatic factors (vascular obstruction, vascular compression, etc.) damage the main supply vessel, the risk of humeral head necrosis increases. Among the traumatic factors, it has been confirmed that proximal humerus fracture damages the blood supply to the humeral head, easily leading to humeral head necrosis.^[5]^ Among nontraumatic factors, long-term use of corticosteroids, alcoholism, hyperuricemia and the genetic disease sickle cell anemia have been shown to cause humeral head necrosis.^[6,7]^ In a few cases, the exact etiology of necrosis was undetermined, and these cases were classified as idiopathic humeral head necrosis.^[8]^ This patient had no history of corticosteroid use, no alcohol abuse, and no family history of related genetic diseases. He did have a history of left shoulder injury and continued to play basketball despite shoulder pain. Because of the lack of original imaging data, according to previous studies, we inferred that the articular cartilage and subchondral bone of the left humeral head may have been damaged during the initial injury, possibly resulting in dissection, and the synovial fluid entered the subchondral space to form cysts, resulting in increased pressure on the left humeral head, obstructing microvascular circulation and healing. The patient continued to play basketball when the left humeral head was not fully healed, and the left humeral head suffered repeated microinjury, which further stripped the articular cartilage and subchondral bone, enlarged the pathological cavity, and increased the inflow of synovial fluid and intraosseous pressure. The microvascular circulation was blocked and aggravated, secondary to ischemic necrosis. Therefore, the T2-weighted image of the patient’s magnetic resonance scan showed that the articular cartilage was discontinuous, the cartilage and the subchondral bone were stripped away, the space between the two contained hyperintensities, and the subchondral bone had collapsed. High and low mixed signal shadows were observed, showing the changes of osteochondritis dissecans (Fig. 1C). Therefore, we believe that the trauma of left humeral head necrosis in this patient was the initial factor, and avascular necrosis secondary to repeated microinjury was the main factor. It is worth noting that this patient was obese and had hyperuricemia, but no urate crystals or gouty bone destruction were found during the operation, and the patient did not have symptoms of multiple joint pain. We did not consider hyperuricemia as a key factor.

According to the Cruess grading system,^[3]^ the patient’s left humeral head necrosis was classified as Stage IV. A recent systematic review on the treatment of humeral head necrosis suggested that conservative treatment should be used for Stage I and II necrosis, and surgical treatment should be recommended for Stages III to V necrosis.^[9]^ Several surgical treatments have been reported, including core decompression, vascularized bone grafting, cartilage grafting, resurfacing, and shoulder arthroplasty, but there are no optimal surgical treatment options for patients with symptomatic humeral head necrosis. Therefore, the overall conditions of the patient should be considered when formulating the surgical plan, including age, body fat index, nature of work, and future progress. In addition, research on the effects of surgical treatment of humeral head necrosis is currently limited to a few case reports and short-term follow-up reports. Nakagawai et al^[10]^ performed autologous iliac bone grafting in a 33-year-old woman with idiopathic necrosis of the left humeral head and achieved satisfactory results at the 2-year follow-up. Autologous cartilage transplantation (mosaicplasty) is often used to treat ankle and knee cartilage defects.^[11]^ Since the development of the surgery, it has also been used to treat humeral head necrosis caused by corticosteroids and has achieved good results in a short period of time. Hotta et al^[12]^ performed autologous cartilage transplantation in a 20-year-old man with steroid-induced right humeral head necrosis. At the 1.5-year follow-up, the patient had no pain, an improved range of motion, and no progression of right humeral head necrosis. Hasegawa et al^[13]^ performed autologous cartilage transplantation for a 53-year-old female patient with steroid-induced humeral head necrosis and achieved good clinical and imaging results at the 2-year follow-up. In that case, the collapsed area was approximately 400 mm^2^, which is the largest area of necrosis reported thus far. In our case, the patient was young, and the cause of necrosis was considered to be secondary ischemic necrosis caused by repeated microinjury. The necrosis was Stage IV, and the necrosis area was large (576 mm^2^). Studies have shown that using joint replacement to treat Stage IV and Stage V necrosis can obtain good results,^[14]^ but we considered that the patient is younger, and joint replacements include inherent risks, such as prosthesis lifespan and infection. At the same time, it has been reported in the literature in recent years that autologous cartilage transplantation can achieve good results,^[12,13]^ so we finally chose to preserve the joints. The patient was treated with left humeral head lesion removal and autologous osteochondral transplantation. Unfortunately, we did not perform biopsy pathology during the operation. According to the analysis of current literature reports, for this patient, arthritis may occur prematurely in the future, and shoulder joint replacement may be needed, but the surgery we performed may delay or even avoid the occurrence of arthritis. Our method may currently be the best surgical treatment plan, although long-term follow-up is required to evaluate the surgical effect.

## 4. Conclusion

Non-drug-induced humeral head necrosis is rare. Autologous osteochondral transplantation is currently one of the most mature and effective treatment methods. The short-term curative effects for this patient are satisfactory, but the patient is young and has a large collapsed area, so long-term follow-up is worthwhile.

## Author contributions

**Conceptualization:** Daohong Zhao.

**Data curation:** Yongsheng Liu, Zhaowei Jiang.

**Funding acquisition:** Daohong Zhao.

**Investigation:** Duo Shen.

**Methodology:** Jia Zhong, Duo Shen.

**Software:** Jia Zhong.

**Writing – original draft:** Yongsheng Liu, Duo Shen.

**Writing – review & editing:** Zhaowei Jiang.

## References

[1] Sarris I Weiser R Sotereanos DG. Pathogenesis and treatment of osteonecrosis of the shoulder. Orthop Clin North Am. 2004;35:397–404, xi. 15271548 10.1016/j.ocl.2004.03.004

[2] Kircher J Patzer T Ziskoven B. Arthroscopically assisted retrograde drilling of the humeral head with a guiding device. Knee Surg Sport Tr A. 2015;23:1442–6. 10.1007/s00167-013-2783-6 24296988

[3] Cruess RL. Experience with steroid-induced avascular necrosis of the shoulder and etiologic considerations regarding osteonecrosis of the hip. Clin Orthop Relat Res. 1978;86:93. 639411

[4] Meyer C Alt V Hassanin H. The arteries of the humeral head and their relevance in fracture treatment. Surg Radiol Anat. 2005;27:232–7. 15999218 10.1007/s00276-005-0318-7

[5] Patel S Colaco HB Elvey ME. Post-traumatic osteonecrosis of the proximal humerus. Njury. 2015;46:1878–84. 10.1016/j.injury.2015.06.026 26113032

[6] Hasan SS Romeo AA. Nontraumatic osteonecrosis of the humeral head. J Shoulder Elb Surg. 2002;11:281–98. 10.1067/mse.2002.124347 12070505

[7] Mont MA Payman RK Laporte DM. Atraumatic osteonecrosis of the humeral head. J Rheumatol. 2000;27:1766–73. 10914865

[8] Cicak N Pećina M Daković M. Idiopathic osteonecrosis of the humeral head. Acta Med Croatica. 1995;49:93–8. 7580046

[9] Franceschi F Franceschetti E Paciotti M. Surgical management of osteonecrosis of the humeral head: a systematic review. Knee Surg Sport Tr A. 2017;25:3270–8. 10.1007/s00167-016-4169-z 27198139

[10] Nakagawa Y Ueo T Nakamura T. A novel surgical procedure for osteonecrosis of the humeral head: reposition of the joint surface and bone engraftment. Arthroscopy. 1999;15:433–8. 10355720 10.1016/s0749-8063(99)70062-9

[11] Hangody L Dobos J Baló E. Clinical experiences with autologous osteochondral mosaicplasty in an athletic population: a 17-year prospective multicenter study. M J Sport Med. 2010;38:1125–33. 10.1177/0363546509360405 20360608

[12] Hotta T Kozono N Takeuchi N. Steroid-induced osteonecrosis of the humeral head in a 20-Year-old man treated with an osteochondral autograft: a case report. Mod Rheumatol Case Rep. 2022. 10.1093/mrcr/rxac037 35460258

[13] Hasegawa A Mihata T Shimizu H. Osteochondral autograft transplantation for the treatment of steroid-induced osteonecrosis of the humeral head: a case report. J Shoulder Elb Surg. 2021;30:e76–83. 10.1016/j.jse.2020.08.033 32920108

[14] Schoch BS Barlow JD Schleck C. Shoulder arthroplasty for atraumatic osteonecrosis of the humeral head. J Shoulder Elb Surg. 2016;25:238–45. 10.1016/j.jse.2015.07.019 26350879

